# Thermochemical recycling of waste disposable facemasks in a non-electrically powered system

**DOI:** 10.1007/s44242-023-00010-w

**Published:** 2023-04-04

**Authors:** Kingsley O. Iwuozor, Ebuka Chizitere Emenike, Agbana Abiodun Stephen, Otoikhian Shegun Kevin, Joy Adeleke, Adewale George Adeniyi

**Affiliations:** 1 Department of Pure and Industrial Chemistry, Nnamdi Azikiwe University, P. M. B. 5025, Awka, Nigeria; 2 Department of Chemical Engineering, University of Ilorin, P. M. B. 1515, Ilorin, Nigeria; 3 Edo State University, Uzairue, Nigeria; 4 Chemical Engineering Department, Landmark University, Omu-Aran, Nigeria

**Keywords:** Biochar, Biomass, Covid-19, Facemask, Thermochemical conversion, 生物炭, 生物质, Covid-19, 口罩, 热化学转换

## Abstract

The COVID-19 pandemic encouraged the use of plastic-based personal protective equipment (PPE), which aided greatly in its management. However, the increased production and usage of these PPEs put a strain on the environment, especially in developing and underdeveloped countries. This has led various researchers to study low-cost and effective technologies for the recycling of these materials. One such material is disposable facemasks. However, previous studies have only been able to engage electrically powered reactors for their thermochemical conversion, which is a challenge as these reactors cannot be used in regions with an insufficient supply of electricity. In this study, the authors utilized a biomass-powered reactor for the conversion of waste disposable facemasks and almond leaves into hybrid biochar. The reactor, which is relatively cheap, simple to use, environmentally friendly, and modified for biochar production, is biomass-powered. The co-carbonization process, which lasted 100 min, produced a 46% biochar yield, which is higher than previously obtained biochar yields by other researchers. The biochar thus obtained was characterized to determine its properties. FTIR analysis showed that the biochar contained functional groups such as alkenes, alkynes, hydroxyls, amines, and carbonyls. The EDX analysis revealed that the biochar was primarily made of carbon, tellurium, oxygen, and calcium in the ratios of 57%, 19%, 9%, and 7%, respectively. The inclusion of the facemask decreased the surface area and porosity of the biochar material, as evidenced by its surface area and pore characteristics.

## Abstract (zh)

2019年新冠病毒肆虐，为更好的防护和疫情管理，人们使用了大量的塑料基个人防护用品 (PPE) 。然而，大量生产和使用个人防护用品给环境 造成巨大的压力，特别对发展中国家和不发达国家。因此，许多科研人员研究如何有效且低成本回收这些废弃材料的 技术，例如针对一次性口罩。以前的研究聚焦于使用电力反应堆进行热化学转换，然而这些反应堆不能在电力供应不 足的地区使用。在本文中，作者利用生物质动力反应器将废弃的一次性口罩和杏树叶转化为混合生物炭。该反应器由 生物质提供燃料动力，相对便宜、使用简单、环保，并可经过改造后制备生物炭。持续100分钟的共同燃烧碳化过程可 得到46%的生物炭产率，高于其他研究人员获得的生物炭产率。随后，对得到的生物炭进行表征以确定其性能。红外光 谱分析表明，生物炭中含有烯烃、炔烃、羟基、胺、羰基等官能团。能量色散X射线光谱分析表明，生物炭主要由碳、 碲、氧和钙组成，其含量分别为57%、19%、9%和7%。加入废弃的一次性口罩共同燃烧制备生物炭，能有效降低制备 而成的生物炭材料的表面积和孔隙率。

## Introduction

In the year 2019, the deadly coronavirus disease (COVID-19) ravaged the entire planet with a fatality rate of more than five million deaths [1, 2]. To assist in battling the pandemic, the use of plastic-based personal protective equipment (PPEs) such as hand sanitizer bottles, medical suits, gloves, and facemasks was encouraged, as well as social distancing [3]. Even though an increase in the production of these PPEs helped curtail the spread of the virus, the used or contaminated material began to constitute an environmental menace in the long run, especially in underdeveloped and developing nations with weak environmental policies and implementation. Various technologies have been utilized for the management of the waste PPEs, with thermal treatment topping the list as the high temperature deactivates and neutralizes the virus. One such method is incineration [4]. However, the shortcomings of this method are that even though it gives room for energy recovery from the waste plastics as used in some developed and developing nations, the incineration of some plastics like polyvinyl chloride (PVC) produces various harmful chlorine-based compounds, and this technique could also contribute to global warming [5]. There is therefore a need for a more environmentally friendly valorization route for these PPEs, such as their thermochemical conversion to biochar. Another merit of using the thermochemical route in comparison with other valorizing techniques is that prior segregation of the material is not required [6].

Globally, it was projected that 3.4 billion disposable facemasks and face shields are thrown away every day, with China topping the list of the most discarded facemasks, followed by India [7]. Facemasks are divided into four different groups, of which the surgical facemasks and the N95 mask, a type of respiratory mask, are the most common [8]. The surgical facemask, which is a type of disposable facemask, was commonly utilized during this period in comparison with the other facemasks due to its efficiency and cost-effectiveness [8–10]. An FTIR study previously performed showed that the layers of the disposable facemask are made up of polypropylene, with the exception of its second layer, which is made up of polyethylene. The ear strap is made up of nylon-6, while the metallic nose wire is composed mostly of iron (Fe) [11].

The thermochemical conversion of disposable facemasks has been studied by various researchers. The pyrolysis of the inner and outer layers of a disposal facemask at three different temperatures has been explored in a bid to produce high-quality bio-oil [12]. Utilizing a microwave-irraidated fast pyrolysis process and together with a corn stover-based biochar catalyst, waste facemasks were thermo-catalytically converted into gas and bio-oil [13]. Using a CO_2_-catalyzed pyrolysis system, disposable facemasks were converted into hydrogen and methane gas [11]. In another study, catalytic gasification was utilized for the thermochemical valorization of waste facemasks [14]. It was observed that, apart from improving hydrogen production, the presence of the nickel-loaded zeolite catalyst can also suppress the formation of hazardous substances during the gasification process [14]. The valorization of discarded facemasks at 700℃ for 2 h to produce mask waste ash catalyst (MWAC) was explored by Kiong, Nordin [15]. The above studies examined the thermochemical conversion of disposable facemasks only.

Thermochemical co-conversion, which involves the thermal degradation of more than one material, is preferred to the conversion of only one material as it can increase the yield of the material [16], is simple and safe [17], is cost-effective as compared to the process of recycling the materials singly [18], and can help decrease different types of waste in the environment in a simultaneous fashion. The thermochemical co-conversion of disposable facemasks has also been studied. For example, the co-hydrothermal liquefaction route has been utilized for the conversion of disposed facemasks and a microalgae (Spirulina platensis) for the production of bio-oil with improved yield [19]. The co-pyrolysis of disposable facemasks and food wastes for the production of fuel-range chemicals was investigated by Park, Choi [20]. Another study engaged the co-hydrothermal liquefaction process for the conversion of the heavy fraction of bio-oil and facemask at temperatures between 700℃ and 900℃ to produce 3D-graphene films, biochar, and bio-oil [21]. The co-conversion of used disposable facemasks and leaves from the almond tree is the focus of this current investigation. The almond tree, which is commonly found on various continents, ranging from Africa to Asia and Australia [22, 23], sheds large quantities of its dried leaves, which are an important component of municipal solid waste in areas where they are found. Even though the fresh leaves of the almond tree have been linked to possessing some medicinal and pharmaceutical applications [24, 25], their dried leaves do not. The dried fallen leaves of almond trees were chosen for this study's feedstock, together with the disposable facemasks, due to their non-competitive use [22, 23].

It can be observed that previous studies on the thermochemical conversion of disposable facemasks involved the use of electrically powered reactors. A limitation to the use of electricity for the conversion process is that it cannot be utilized in rural areas with a low or no source of electricity. This created the need to utilize a reactor that does not depend on electricity to function but on other natural sources of energy. In lieu of this, a biomass-powered top-lit updraft reactor with retort heating, modified for biochar production, was utilized in this study for the co-carbonization of used disposable facemasks and almond leaves. Apart from reducing the cost of conversion of the disposable facemasks into biochar (a cost incurred from electricity), the reactor is also environmentally friendly, simple to use as it doesn’t require much technical knowledge, and very cost-effective in fabrication, which makes its up-scaling in both rural and urban areas very feasible.

## Methodology

### Material preparation

Fallen almond tree leaves were manually collected within the premises of the University of Ilorin. Disposable blue-coloured facemasks were also handpicked around the environs of the university. The nose clips in the facemasks were detached, and the material was cut into small, rectangular pieces (of about 3 cm by 4 cm) with the aid of a pair of scissors. Thereafter, the feedstock, consisting of the facemasks and the almond leaves in the ratio of 1:9, was introduced into the carbonization chamber of the reactor. Delonix regia stems, used as a biomass fuel in this study, were also handpicked around the university premises.

### Carbonization and co-carbonization processes

**Fig. 1:**
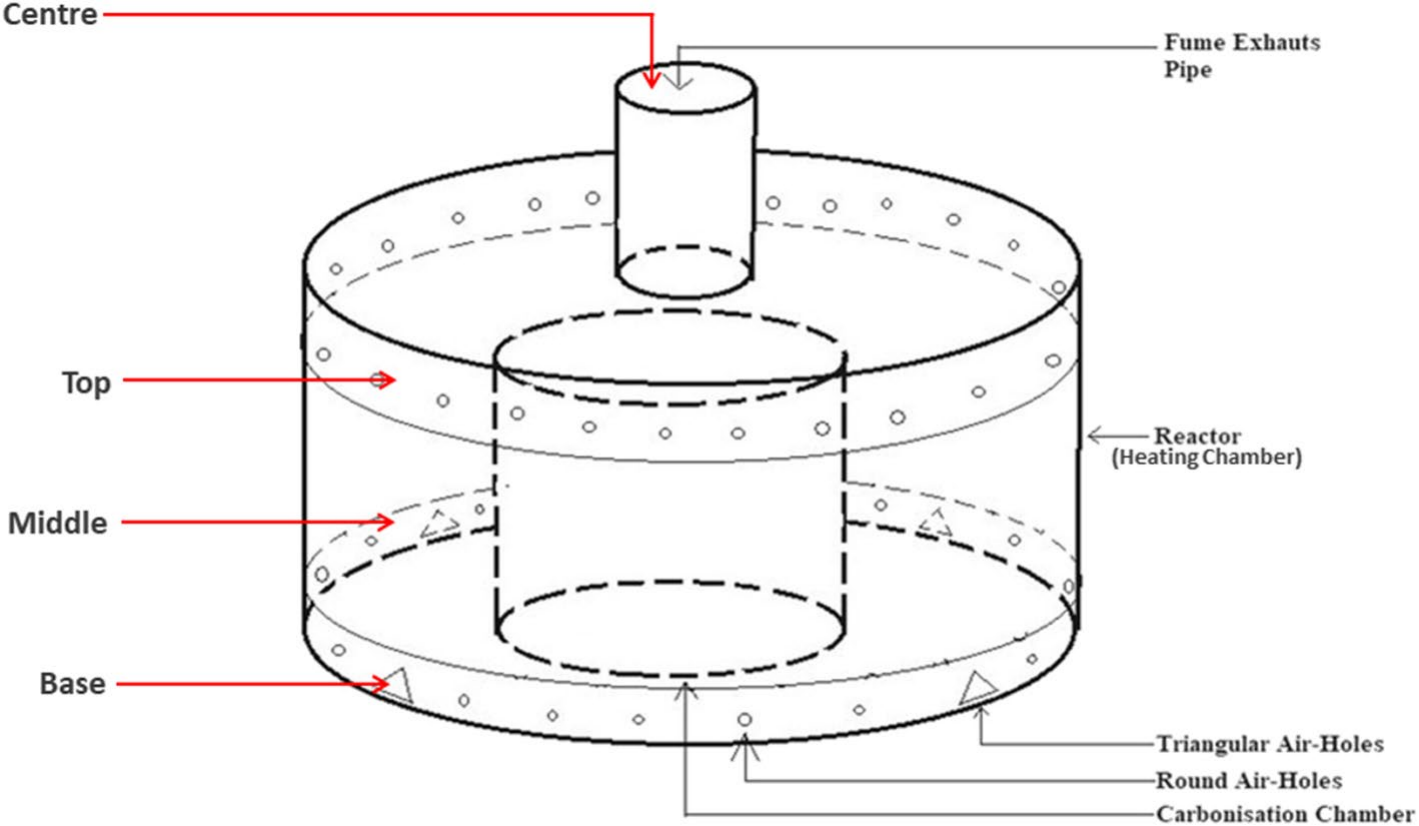
A schematic diagram of the reactor used in this study

The reactor utilized in this study, as shown in Fig. 1, is made up of two chambers: the carbonization chamber and the heating chamber. The former holds a weighed amount of the feed and has a lid that seals it from the top, where the carbonization and co-carbonization process occur. The heating chamber, on the other hand, is also made of aluminum. The heating chamber houses the carbonization chamber along with the fuel biomass.

The reactor, which was operated in an open environment, utilized the top-lit updraft mechanism with retort heating for operation. For the carbonization to take place, the feed materials (facemasks and almond leaves for the co-carbonization process and only almond leaves for the carbonization process) were properly enclosed within the carbonization chamber, which was then properly placed at the centre of the heating chamber. The fuel biomass (Delonix regia stem) of not more than 15 cm was arranged by the side of the carbonization chamber, covering up all empty spaces between the two chambers, till the material filled the heating chamber, just enough to allow it to be properly sealed with its lid. Thereafter, the biomass fuel in the heating chamber was ignited from the top. The system was allowed to burn for 2–3 min after being ignited from the top so as to allow the fire to circulate throughout all parts of the reactor before it was covered with a lid, which gives room for the exhaust of the fumes. This was done in an open environment, and the experiment ended when equilibrium was reached at ambient temperature, when the combustion fuel burned to ash and the feedstock(s) transformed into biochar.

Using a thermometer gun Cason (CA380), the reactor's temperature was measured, and referred to as the temperature at 0 min. The temperature reading was taken at four different vertical points of the reactor, namely, the base, middle, top, and centre. The readings were also taken at two other positions around the reactor, covering an equal distance to obtain triplicate readings. The process of taking the temperature reading was repeated at intervals of ten minutes until the temperature of the reactor was close to room temperature.

After cooling, the carbonization chamber housing the biochar was carefully removed from the heating chamber and then weighed. With the aid of Eq. (1), the yield of the biochar was calculated using the initial weight of the feedstock(s) and the weight of the biochar obtained. The biochar was pulverized and carefully mixed to ensure homogeneity. This material was labeled as ALFB. A similar process (carbonization) was performed on the almond leaves only, and the biochar produced from this process was labeled ALB. The abbreviations ALFB and ALB are used in the remaining part of this manuscript.

1 $$\mathrm{Biochar}\;\mathrm{yield}=\frac{\mathrm{Weight}\;\mathrm{of}\;\mathrm{biochar}}{\mathrm{Weight}\;\mathrm{of}\;\mathrm{initial}\;\mathrm{feedstock}}\times100\%$$

### Characterization

The textural properties of the sample were determined by physically adsorbing N_2_ at 77 K. The total pore volume and the pore size distribution (pore diameter) were computed using the Barrett-Joyner-Halenda (BJH) model, and the specific surface area was determined using the Branauer-Emmett-Teller (BET) equation in the relative pressure range of 0.54—1.17. The morphology and elemental makeup of the monolith were studied using a scanning electron microscope coupled with energy dispersive X-ray spectroscopy (SEM–EDX, Phenom-World BV, Netherlands). Fourier transform infrared spectroscopy (Scimadzu, FTIR-8400S, Japan) was used to elucidate the functional groups present. The spectrum was recorded using the transmittance method in the 4000–650 cm^−1^ region with 30 sample scans.

## Results and Discussion

### Temperature profile and biochar yield

**Fig. 2:**
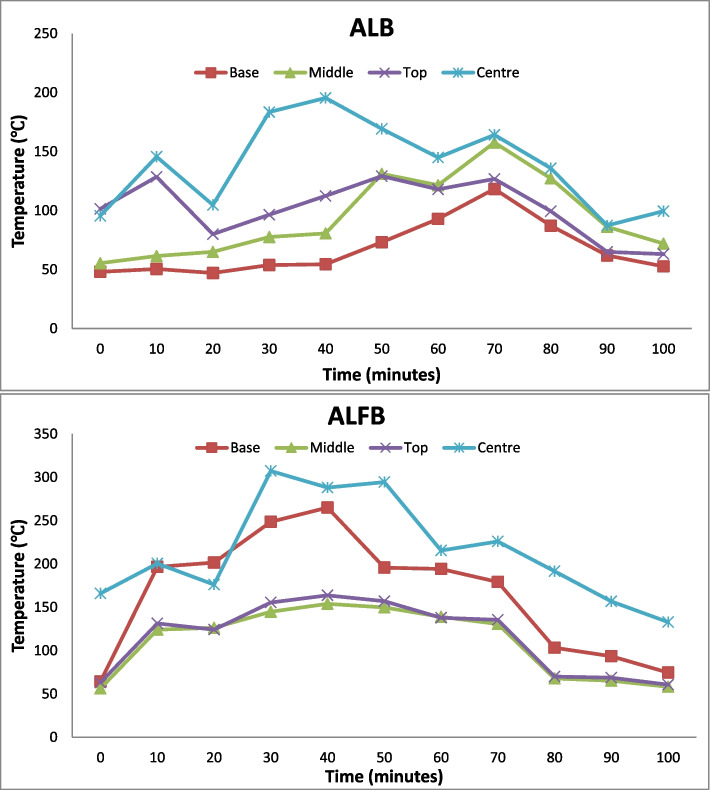
Temperature profile graphs of ALB and ALFB

To further understand the direction of heat flow as well as temperature change in different parts of the reactor, its temperature was measured. This is necessary as it helps us determine the progression of heat as the feed is converted into biochar. The results in triplicate for each region at different temperatures were averaged for the four regions of the reactor and plotted in a graph to give the temperature profile as shown in Fig. 2. The carbonization and co-carbonization systems, which gave rise to ALB and ALFB, respectively, were both allowed to run for a duration of 100 min.

The temperature profile of ALB shows that the temperature of the system increased gradually, spanning the entire reactor, from top to bottom, at time 0 min. This is in line with the working principle of the reactor, as it is termed "top-lit." It is expected that the heat will decrease with an increase in time, and this was observed as time progressed. In addition, as time progressed, it was generally observed that the temperature of the four regions of the reactor increased and reached a peak that was between 40 and 70 min, depending on the region of the reactor. Beyond this peak, a gradual decline in the temperature was observed. The peak temperatures for the reactor's base, middle, top, and center regions were observed at 70 min (118℃), 70 min (157℃), 50 min (129℃), and 40 min (195℃), respectively. This shows that the highest temperature reading was obtained at the centre of the reactor. In addition to this observation, apart from time zero minute, where the temperature at the top was higher than that at the centre, the temperature at the centre was the highest in comparison with other regions for the entire range of reaction duration. This confirms the transfer of thermal energy from the fuel to the inner chamber, as the carbonization chamber is located at the centre of the reactor. Similar findings about the temperature of the core of the reactor have already been made by other researchers, including Ighalo, Onifade and Adeniyi, Ighalo [16].

Just like the carbonization system, the regions of the co-carbonization system experienced an increase in temperature from 0 min up to their peak at 30–40 min, depending on the region of the reactor, after which the temperature decreased gradually. In comparison with the carbonization system, the co-carbonization system experienced higher temperatures, which could be due to the change in the velocity of the wind at the time of the experiment. This is evident as the peak temperatures for the reactor’s base, middle, top, and centre regions were observed at 40 min (265℃), 40 min (153℃), 40 min (163℃), and 30 min (307℃), respectively. Just like the carbonization system, the temperature in the reactor's center was highest within the range of time under study, with the exception of 20 min. Unlike with the carbonization system, where the temperature at the base was greater than that at the middle and top sections of the reactor, the quick increase in temperature at the base was discovered to be obvious throughout the operation. There are two potential causes for this discovery. The first might be caused by the direction and force of the wind, while the second might be brought on by the fuel's packing density surrounding the inner chamber. It is recommended that the density of the biomass fuel be equal in all regions of the reactor for synergistic heating, as the concentration of the fuel in one region could increase the temperature of that region and ensure it burns longer than the other regions. The yields of ALB and ALFB were calculated at the process' completion. ALB was synthesized through the carbonization of almond leaves only, with a yield of 39%. In comparison with other studies, after a carbonization time of two hours, an almond leaf biochar yield of 28% has been previously recorded [26]. For the same carbonization duration, a yield of 14% was achieved for elephant grass biochar [27]. After a duration of 150 min, a biochar yield of 6.9% was obtained for plantain fibres feedstock [28].

Due to these, it was determined that this research's yield was higher than that obtained from carbonizing other biomass feedstocks. From this, it can be concluded that almond leaves are a great feedstock for producing high-yield biochar. ALFB, on the other hand, was produced through the co-carbonization approach, with a yield of 46%. The increase in yield from 39 to 46% could be due to the presence of higher carbon containing polymers in the facemask, which significantly raised the material's yield [29, 30]. In comparison with other studies, a biochar yield of 28.9% at a peak temperature of 200℃ was obtained for the co-hydrothermal liquefaction of disposable facemasks and S. platensis [19]. Another study obtained a yield of 71% for the co-conversion of almond leaves and low-density polyethylene (LDPE) [26]. In other studies, a yield of 62.7% and 45.4% was recorded for the co-carbonization of oil palm fibre and LDPE and sugarcane bagasse and LDPE, respectively [29, 30].

### Analysis of Functional groups

**Fig. 3:**
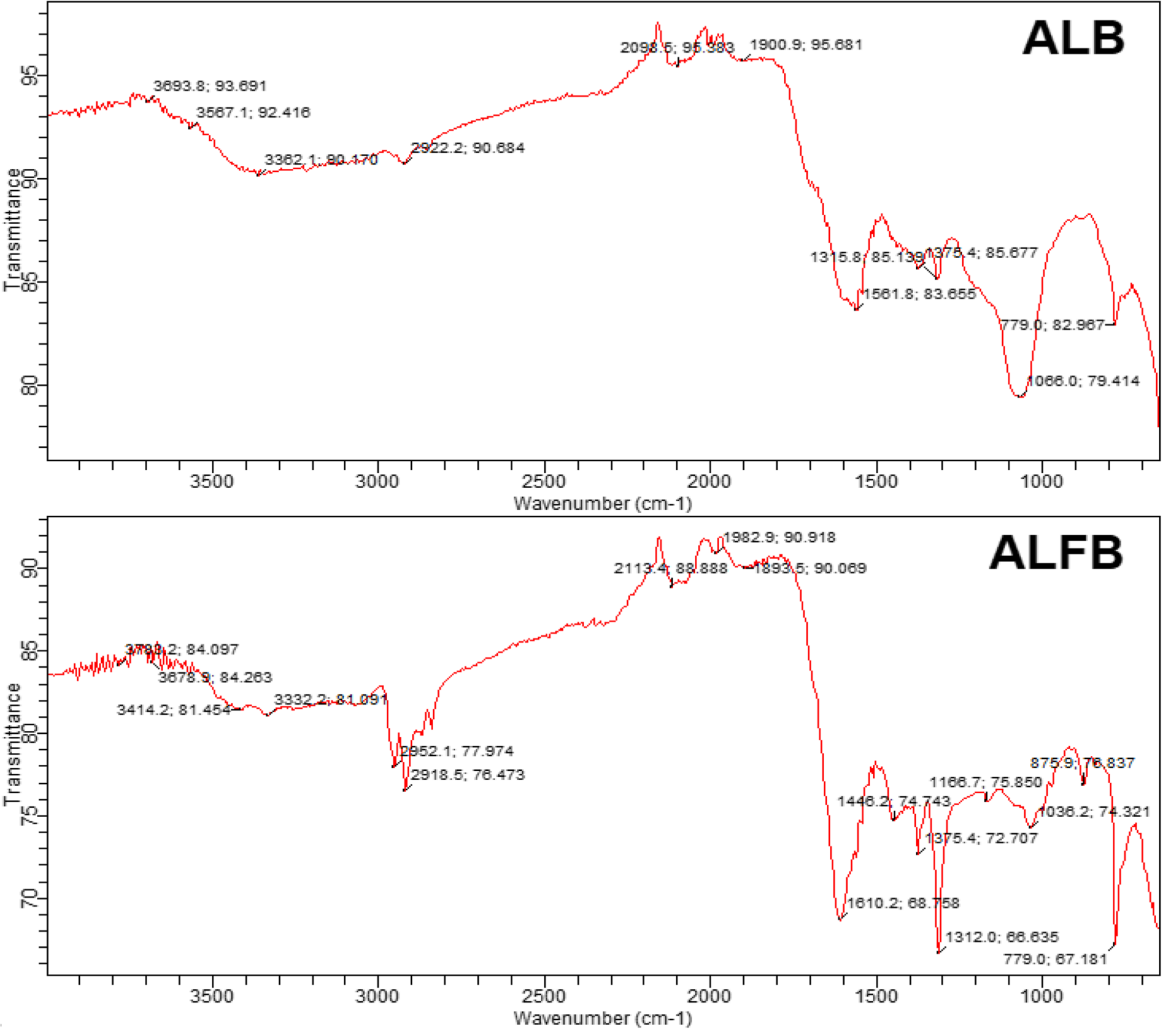
FTIR spectra of ALB and ALFB

FTIR analysis was performed on the biochar to determine its inherent functional groups. By contrasting the spectra of ALFB and ALB obtained from the Fourier transform infrared spectrophotometer, the effect of the facemask on the functional groups of the biochar was investigated, as shown in Fig. 3, and this analysis was based on a number of studies [31–35]. The functional groups of the two materials contain a variety of groups, including the alkene group, the alkyne group, the hydroxyl group, the amine group, and the carbonyl group. Most of these functional groups were observed to be present in both ALFB and ALB, with most of them existing at different yet close peaks in comparison with each other. The presence of the alkene functional group was noted at bands corresponding to 1561.8 cm^−1^ and 1610.2 cm^−1^ for ALB and ALFB, respectively. In addition, the presence of an alkene hydrogen in bending mode was found at 779 cm^−1^ for both ALB and ALFB. Also, the same functional group was present at 875.9 cm^−1^ for ALFB. The extra alkene peak observed in ALFB may have originated from the facemask material, which contains unsaturated polymers such as polyethylene and polypropylene. Another unsaturated carbon functional group, i.e., the alkyne functional group, was observed in both materials. At 2098.5 cm^−1^ and 2113.4 cm^−1^, the -C≡C- was observed. Just like the alkene group, the presence of alkyne hydrogen was also observed at 3362.1 cm^−1^ and 3332.2 cm^−1^ for ALB and ALFB, respectively. This shows that for most of the unsaturated carbon bonds, a shift in peak was observed with the introduction of the disposable facemask material.

Other functional groups, such as the alkane functional group, were observed to exist in the biochars. The peaks at 2923.2 cm^−1^ and 2952.1 cm^−1^ for ALB and ALFB, respectively, were assigned to the carbon-hydrogen stretching vibrations of aliphatic groups [36]. The bands between 3693.8 cm^−1^ and 3567.1 cm^−1^ for ALB and 3788.2 cm^−1^ and 3678.9 cm^−1^ for ALFB were assigned to the -OH functional group [37, 38]. In addition, the C-O symmetric stretch of cellulose was found to exist at a peak of 1066.0 cm^−1^ for ALB and 1166.7 cm^−1^ and 1036.2 cm^−1^ for ALFB. The vibrating peaks at 1375.4 cm^−1^ and 1312.0 cm^−1^ were assigned to the presence of the C-O–H bend. Similar bands were also noted in ALB at 1375.4 cm^−1^ and 1315.8 cm^−1^. The existence of a new peak in the spectra of ALFB at 3414 cm^−1^, which didn’t appear at ALB, was observed. This peak, which shows the presence of a nitrogen–hydrogen stretch of an amine or amide functional group, was transferred from the facemask into the biochar. The presence of this group makes ALFB more suitable to be used as an additive in the soil, as it has the capacity to transfer its nitrogen content into the soil.

In conclusion, comparing the spectra of ALFB and ALB revealed a significant peak shift representing the same functional group. This phenomenon, which is caused by the introduction of the facemask material, is not new, as some researchers have obtained similar results [3, 19]. In addition, some peaks (1375.4 cm^−1^ and 779.0 cm^−1^) were found to be present in both materials. These peaks, together with those exhibiting peak shifts, could be said to have originated from the almond leaves. Another observation made was the introduction of new peaks in ALFB that are absent in ALB. Such bands at 3414.2 cm^−1^, 2952.1 cm^−1^, 1166.7 cm^−1^, and 875.9 cm^−1^ could be said to have been introduced by the facemask into the biochar. There is no doubt that ALFB contains more functional groups in comparison with ALB. This invariably makes it a better adsorbent material than ALB. Therefore, it is sufficient to state that the FTIR spectra show the presence of the facemask composition in ALFB.

### Chemical Composition of ALFB and ALB

The energy dispersive X-ray spectroscopy point analysis was used to investigate the elemental analysis of ALFB and ALB. The result, which is summarized in Table 1, shows that the two materials are primarily composed of carbon. Previous studies have also shown that the biochar generated from almond leaves contained carbon as its primary inorganic ingredient [39]. However, the carbon content decreased from 61.16% to 57.21% once the facemask was introduced. Even though the facemask that was introduced was primarily composed of carbon, the observed decrease could have resulted from the production of carbon-based non-condensable gases such as CO and CO_2_ during the carbonization process. However, this finding contradicts the findings of the bulk of previous investigations, which found a rise in carbon content since carbon and hydrogen are the principal elemental components of facemasks [29, 30]. Nevertheless, the biochar samples have a high concentration of carbon, meaning that they may be used to sequester carbon and reduce greenhouse gas emissions [40, 41].

**Table 1:** Table showing the elemental make-up of ALB and ALFB

Element	ALB		ALFB	
	Atomic Conc	Weight Conc	Atomic Conc	Weight Conc
Carbon	74.39	61.16	81.27	57.21
Oxygen	19.27	21.11	10.36	9.72
Calcium	2.52	6.92	3.38	7.94
Silver	0.72	5.29	-	-
Silicon	1.92	3.69	0.48	0.80
Fluorine	0.54	0.70	-	-
Aluminum	0.31	0.58	0.20	0.32
Magnesium	0.33	0.54	0.33	0.46
Tellurium	-	-	2.58	19.27
Iron	-	-	0.50	1.63
Copper	-	-	0.43	1.62
Potassium	-	-	0.38	0.86
Chlorine	-	-	0.08	0.17

After carbon, the next most abundant elements in ALB and ALFC were observed to be oxygen and tellurium, respectively. This implies that the introduction of the facemask into the system reduced the concentration of oxygen in ALB from 21.11% to 9.27% and introduced 19.27% of tellurium into the material, which is absent in ALB. In addition, other elements apart from calcium also observed a decrease with the introduction of the facemask as feed. Among the elements, silicon decreased from 3.69% to 0.80%, magnesium decreased from 0.54% to 0.46%, aluminum decreased from 0.58% to 0.32%, and silver and fluorine decreased from 5.29% and 0.7%, respectively, to 0%. However, it is evident that the facemask material introduced several other elements into the biochar material. Elements in this group include iron, copper, potassium, and chlorine. In addition, it led to an increase in the concentration of calcium, from 6.92% to 7.94%.

### Surface morphological properties

The surface morphology of the biochars was studied with the aid of scanning electron microscopy (SEM). Table 2 displays the SEM micrographs of ALB and ALFB at two different resolutions, namely × 500 and × 1500, respectively. At × 500 resolution, the surface morphology of ALB shows the existence of a sizable crack that divides the biochar into two. Of the two, one has unevenly spaced silvery patches covering its surface. The EDX finding supports the theory that these silvery spots could be the result of calcium and silver deposits on ALB's surface [42]. Contrarily, the second half was seen to be smoother and feature fewer discernible silvery patches than the first. The homogeneity of the silvery patches was seen to have increased at higher resolution (× 1500) compared to the reduced magnification. ALB's surface is often depicted as having a rough texture, which might perform well as a soil catalyst and addition to prevent nutrient and water loss from the soil.

**Table 2:**
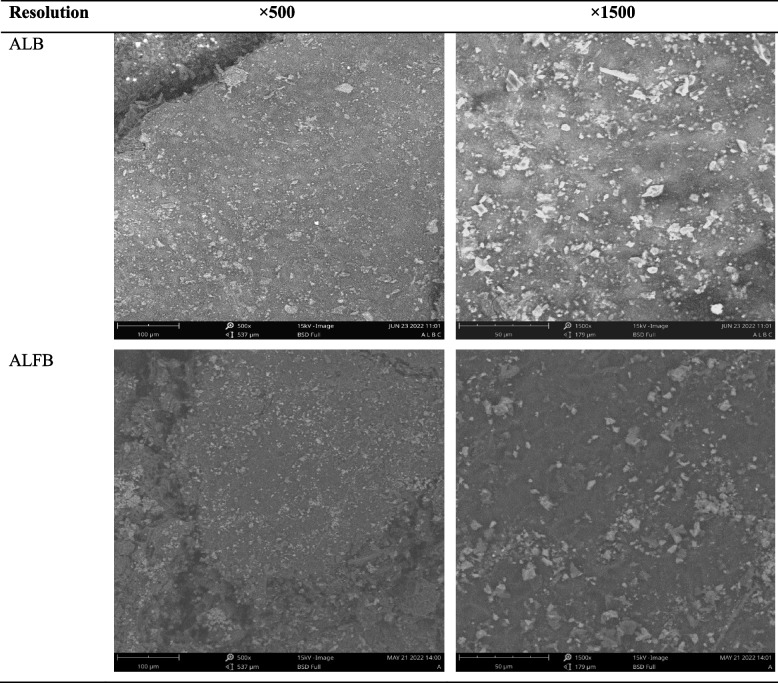
SEM micrographs of the biochars at two different resolutions

At low resolution, the micrograph of ALFB shows the presence of a smoother surface in comparison with ALB. The presence of metallic deposits can also be observed, distributed in a homogeneous fashion on its surface [16]. Furthermore, the presence of some crevices was observed on its surface; however, they were not evenly distributed. At high magnification, the metallic deposits were more pronounced on the surface of ALFB, which is homogenously distributed.

### Surface area and pore characteristics

The porosity and surface area of the biochars were studied using various methods, i.e., Langmuir, BET, BJH, DR, DA, and DFT. Table 3 shows that the surface area of ALFB is less than that of ALB. This observation is demonstrated by the surface areas determined by methods such as BET, Langmuir, BJH, and DFT, which obtained surface areas of 398.5 m^2^/g and 185.9 m^2^/g, 6890 m^2^/g and 971.8 m^2^/g, 412.4 m^2^/g and 213 m^2^/g, 84.9 m^2^/g and 44.8 m^2^/g for ALB and ALFB, respectively. This implies that the inclusion of the facemasks on the almond leaves decreased the surface area of the biochar. Previous experiments on co-carbonization systems have shown similar results [26, 29].

**Table 3:** Pore and surface area characteristics of the biochars

Methods	Properties	ALB	ALFB
BET	Surface area (m^2^/g)	398.500	185.979
DA	Micropore volume (cc/g)	0.355	0.169
	Pore diameter (nm)	3.000	2.920
BJH	Surface area (m^2^/g)	412.457	213.075
	Pore volume (cc/g)	0.203	0.104
	Pore diameter (nm)	2.138	2.131
Langmuir	Surface area (m^2^/g)	6890.821	971.856
DR	Average pore width (nm)	6.516	6.181
	Micropore volume (cc/g)	0.144	0.072
	Micropore surface area (m^2^/g)	405.115	201.468
DFT	Pore volume (cc/g)	0.103	0.054
	Surface area (m^2^/g)	84.931	44.857
	Average pore width (nm)	2.647	2.647

Just like the trend observed for the surface area, the pore properties of ALB were also observed to be greater than those of ALFB, as seen in Table 3, indicating that ALFB is less porous than ALB. This is evident in the micropore volume calculated using the DA and DR methods, the pore diameter calculated using the DA and BJH methods, the pore volume calculated using the BJH and DFT methods, and the average pore width calculated using the DR and DFT methods. This finding is consistent with that obtained by the SEM micrographs in Sect. 3.4. The reason for the increase in porosity of the ALB could be due to the volatilization of the leaves at high temperatures, which increases its pore properties. However, the presence of the polymers in the facemask reduced the rate of volatilization [43]. As shown by the DA and BJH methods, the pore diameters of both biochar samples are in the mesoporous region. Mesoporous materials have diameters ranging from two to fifty nanometers. Mesoporous materials have a wide range of uses, including drug delivery, solar cells, and nucleic acid detection [44, 45].

## Conclusion

This work produced biochar in a non-electrically driven reactor by co-carbonizing waste facemasks and almond leaves at a feedstock ratio of 1:9. The reactor, which operates on the principle of top-lit updraft technology with retort heating and is powered by biomass fuel as an alternative to electricity, is modified for biochar production. Biochar was also produced with the use of only the almond leaves (as a control). The feedstock was carbonized and co-carbonized for a period of one hour and forty minutes, after which the co-carbonized biochar was observed to possess 7% more yield than the carbonized biochar. Based on the characterization done on the biochar, the following conclusions were obtained:

1. The biochar contains functional groups such as alkenes, alkynes, hydroxyls, and carbonyls, according to FTIR analysis. In addition, it was observed that the co-carbonized product contained more functional groups than the carbonized product.
2. The EDX findings demonstrated that adding the facemask to the feedstock reduced its carbon and oxygen concentration in the biochar but introduced other elements like tellurium and iron.
3. The presence of the facemask reduced the surface area and porosity of the biochar material.
4. This study shows that facemasks can be thermochemically recycled into biochar in a non-electrically powered reactor, which is relatively cheap, environmentally friendly, and simple to use and maintain, and the resulting biochar can be utilized for environmental applications such as carbon sequestration and greenhouse gas reduction, as well as agricultural applications to improve soil conditions.

## Data Availability

The raw data for this research are available by contacting the corresponding authors.
